# Differential immune gene response in gills, skin, and spleen of rainbow trout *Oncorhynchus mykiss* infected by *Ichthyophthirius multifiliis*

**DOI:** 10.1371/journal.pone.0218630

**Published:** 2019-06-20

**Authors:** Khairul Syahputra, Per W. Kania, Azmi Al-Jubury, Huria Marnis, Agung Cahyo Setyawan, Kurt Buchmann

**Affiliations:** Department of Veterinary and Animal Science, Faculty of Health and Medical Sciences, University of Copenhagen, Frederiksberg C, Denmark; INRA, FRANCE

## Abstract

Infection of rainbow trout with the parasitic ciliate *Ichthyopthirius multifiliis* induces differential responses in gills, skin and spleen. A controlled experimental infection was performed and expression of immune-relevant genes in skin, gills, and spleen were recorded by qPCR at day 1 and 8 after parasite exposure. Infection induced a marked reaction involving regulation of innate and adaptive immune genes in rainbow trout at day 8 post-infection. The expression level of a total of 22 out of 24 investigated genes was significantly higher in gills compared to skin reflecting the more sensitive and delicate structure of gills. Especially pro-inflammatory cytokines IL-6, IL-17 C1, regulatory cytokines IL-4/13A, IL-10, TGFβ, complement factor C5, chemokines CK10, CK12, acute phase proteins (precerebellin, hepcidin) and immunoglobulins (IgM, IgT) displayed differential expression levels. The spleen, a central immune organ with no trace of the parasite, showed elevated expression of IgM, IgT, complement factor C5 and chemokine CK10 (compared to skin and gills directly exposed to the parasite), indicating an interaction between the infected surface sites and central immune organs. This communication could be mediated by chemokines CK10 and CK12 and cytokine IL-4/13A and may at least partly explain the establishment of a systemic response in rainbow trout against the parasite.

## Introduction

The parasitic ciliate *Ichthyophthirius multifiliis* is one of the most problematic parasites in aquaculture affecting a wide range of different freshwater fish species worldwide [1]. Rainbow trout (*Oncorhynchus mykiss*) is one of the main aquaculture species [2, 3] suffering from infections. A series of investigations have documented that fish hosts respond to infection with a protective immune response [4–11] but how the host regulates the different parasitic stimulations of the fish body surfaces and eventually establishes a systemic immunity is unknown. The present study addressed this issue by measuring immune reactions in rainbow trout surfaces and central immune organs at an early and late time point during *I*. *multifiliis* infection. Expression of a total of 24 immune genes (encoding cytokines, chemokines, complement factors, acute phase proteins, immune cell receptors, and immunoglobulins) were investigated in mucosal surfaces (gills, skin) and a central organ (spleen). The study thereby contributes to the understanding of how infections in the surface of a fish may elicit a protective systemic response.

## Materials and methods

### Ethics statement

The infection experiments were conducted at the Laboratory of Aquatic Pathobiology fish infection facilities at the University of Copenhagen (Frederiksberg C, Denmark). Animal care and investigations were performed according to license 2013-15-0201-00764a (The Experimental Animal Inspectorate under the Ministry of Food, Agriculture and Fisheries).

### Fish and rearing conditions

Fish handling and infection procedures in this work were described previously [12]. Briefly, rainbow trout fry were produced and reared under pathogen-free conditions in a recirculated closed system (Bornholm Salmon hatchery, Nexø, Denmark). Fish were then transported to our experimental rainbow trout facility and kept in 200 L fish tanks (water temperature of 12–14°C) until initiation of the experiment. The experimental fish (body weight mean (SD): 7 (2) g) were acclimatized in 4 x 60 L tanks (20 fish in each) containing freshwater (municipal tap water, Frederiksberg) equipped with internal biofilters (Eheim, Germany), plastic plants (enrichment), and continuous aeration using air stones for two weeks before initiation of the infection experiment. Fish were fed commercial pelleted feed (Biomar, Denmark) every second day (1% of biomass). The aquaria were covered using a screen of dark plastic to avoid influence of stressors (light, movements) from the exterior. Rearing water temperature was set to 15–16°C and water quality (NH_3_, NO_2_, and pH) was monitored with standard test kits (Merck, Germany) throughout the experimental period.

### Infection procedure and sampling

The experimental rainbow trout were randomly divided into two groups (infected/non-infected), each comprising two replicates. For the infection group, fish were exposed to infective theronts by adding a solution of parasites (2,400 theronts/fish). Theronts were produced from tomocysts developed from tomonts released from infected fish according to standard procedures [7, 13]. For the control group, the same treatments were conducted but the corresponding tanks were sham-infected by pouring a similar volume of pure water into the tanks.

A total of 5 fish from each tank were randomly sampled at day 1 and 8 post-infection (dpi). Fish were collected by a hand-net and subsequently euthanized in MS-222 (Cat. no. A5040, Sigma-Aldrich) (300 mg/L) followed by tissue collection. Tissues (skin, gills, and spleen) were aseptically sampled and immediately placed into 2 mL tubes containing RNAlater (Cat. no. R0901, Sigma Aldrich), pre-stored at 4°C for 24 h and then stored at -20°C until conducting gene expression analysis.

### RNA extraction, cDNA synthesis and real-time PCR

Total RNA was extracted from tissue samples using GenEluteTM kit (Cat. no. RTN350-1KT, Sigma-Aldrich), according to the manufacturer’s instruction and subsequently DNase treated with DNase I (Cat. no. ENO521, Thermo Scientific). The quantity and purity of RNA were measured at 260/280 nm (NanoDrop 2000 Spectrophotometer, Thermo Scientific) and DNAse efficacy and RNA integrity were evaluated by electrophoresis on 1% agarose gels with ethidium bromide (EtBr) staining.

The first-strand cDNAs were synthesized using 1000 ng of total RNA, MultiScribe Reverse Transcription reagent (Thermo Fisher Scientific), and random hexamers (Roche) in a 20 μl setup. The reaction was placed at 37°C for 60 min in a Thermal Cycler (T100^TM^ Thermal Cycler, BioRad). Subsequently, the synthesized cDNA was stored at -20°C until further use.

Presence of trophonts in skin and gills were confirmed visually under the dissection microscope (magnification x 7–40) but a quantitative estimation of the parasite burden was obtained by performing quantitative real-time PCR (qPCR) with specific primers and probes for the gene encoding the parasite’s I-antigen. Likewise the expression of immune-associated genes was evaluated by qPCR assays using the synthesized cDNA with specific primers and corresponding probes listed in S1 Table. The qPCR reactions were carried out in a 96 well plate containing 5 ng/μl of cDNA, 6.25 μl of Brilliant® II QPCR master mix (Agilent Technologies), forward and reverse primer (0.8 μM each), and TaqMan® probe (0.4 μM) in a total volume reaction of 12.5 μl. The reactions were performed on an AriaMx Real-Time PCR system (Agilent Technologies) under the following conditions: 95°C for 15 min followed by 45 cycles of 95°C for 10 s and 60°C for 45°C. The endogenous reference gene (EF1-α) was used to normalized the relative expression of the target genes.

### Data analysis

The 2^-ΔΔCt^ method was applied to determine the relative gene expression presented as the fold increase or decrease of the infected group relative to the time point control groups (mean expression level adjusted to 1). The absence of tank effect was tested and confirmed before pooling the gene expression data from duplicate tanks. To account for biological variation, only gene regulations with at least 2-fold change were considered significant. The statistical difference between groups was determined using a Student’s *t-*test applying a probability level of 5% (p<0.05). Additionally, correlations among the gene expression levels in investigated tissues at different time-points were analyzed by using Pearson correlation. The relative gene expression was visualized using a heat map, generated using gplots (v3.01) in R (v.3.44).

## Results

### The presence of parasites in infected tissues

**Fig 1:**
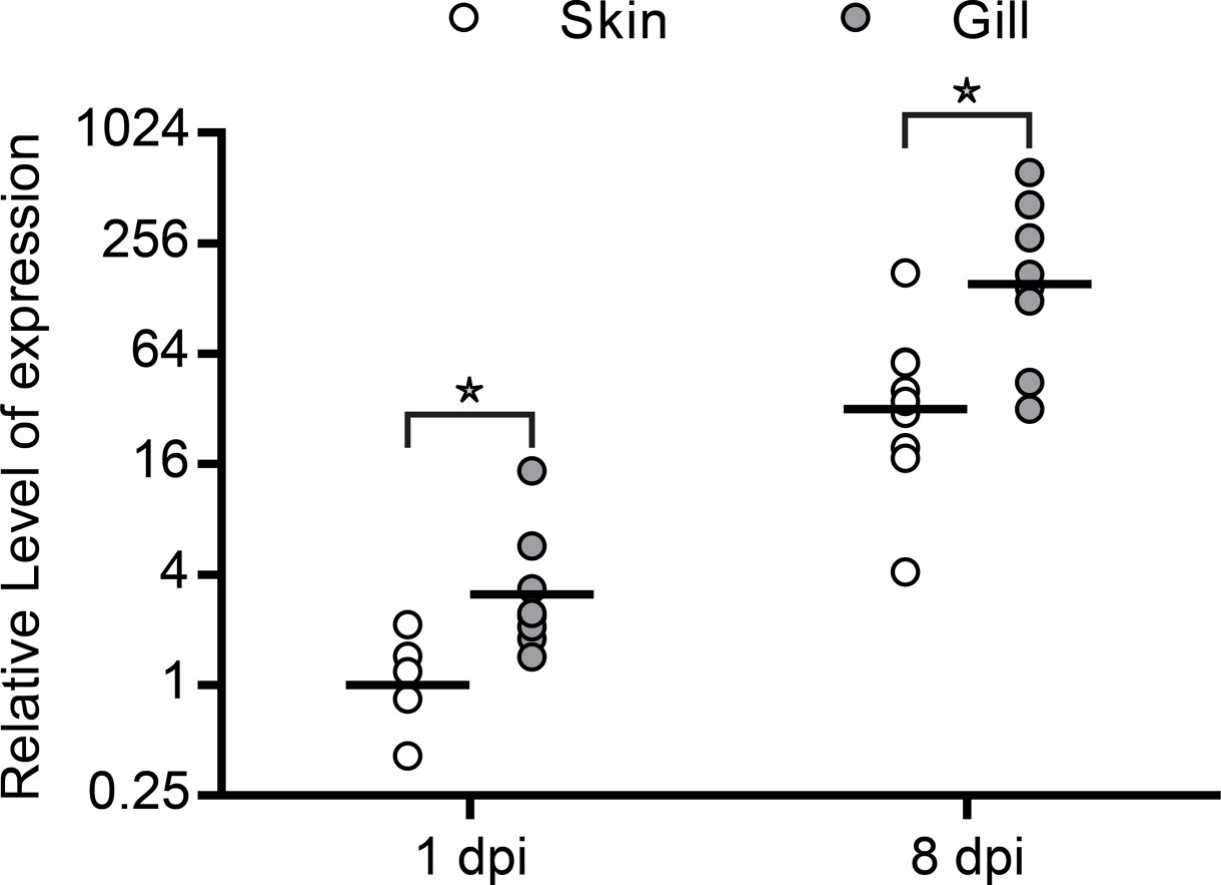
Relative levels of *I*. *multifiliis* is represented relative to the reference gene EF1α by calculating 2^-ΔCq^ normalized to the group having the lowest expression level, skin at 1 dpi. Student t-test (p<0.05) showed that gills had significantly higher level of *I*. *multifiliis* at both 1 dpi and 8 dpi (3.1 and 4.8 fold, respectively). No transcription of the gene was recorded in spleen.

Presence of parasites in all examined tissues was confirmed by microscopy and quantifying the expression level of the gene encoding the specific *Ichthyophthirius multifiliis* I-antigen. The parasite gene transcript was measured in infected skin and gills at both day 1 and 8 after infection, but no expression was found in spleen. The transcript level in gills was higher when compared to skin at both time-points (3.1 and 4.8 fold, respectively) and the expression levels of I-antigen were increasing in both infected tissues indicating increased transcription during the experiment (Fig 1).

### Expression of immune-relevant genes

**Fig 2:**
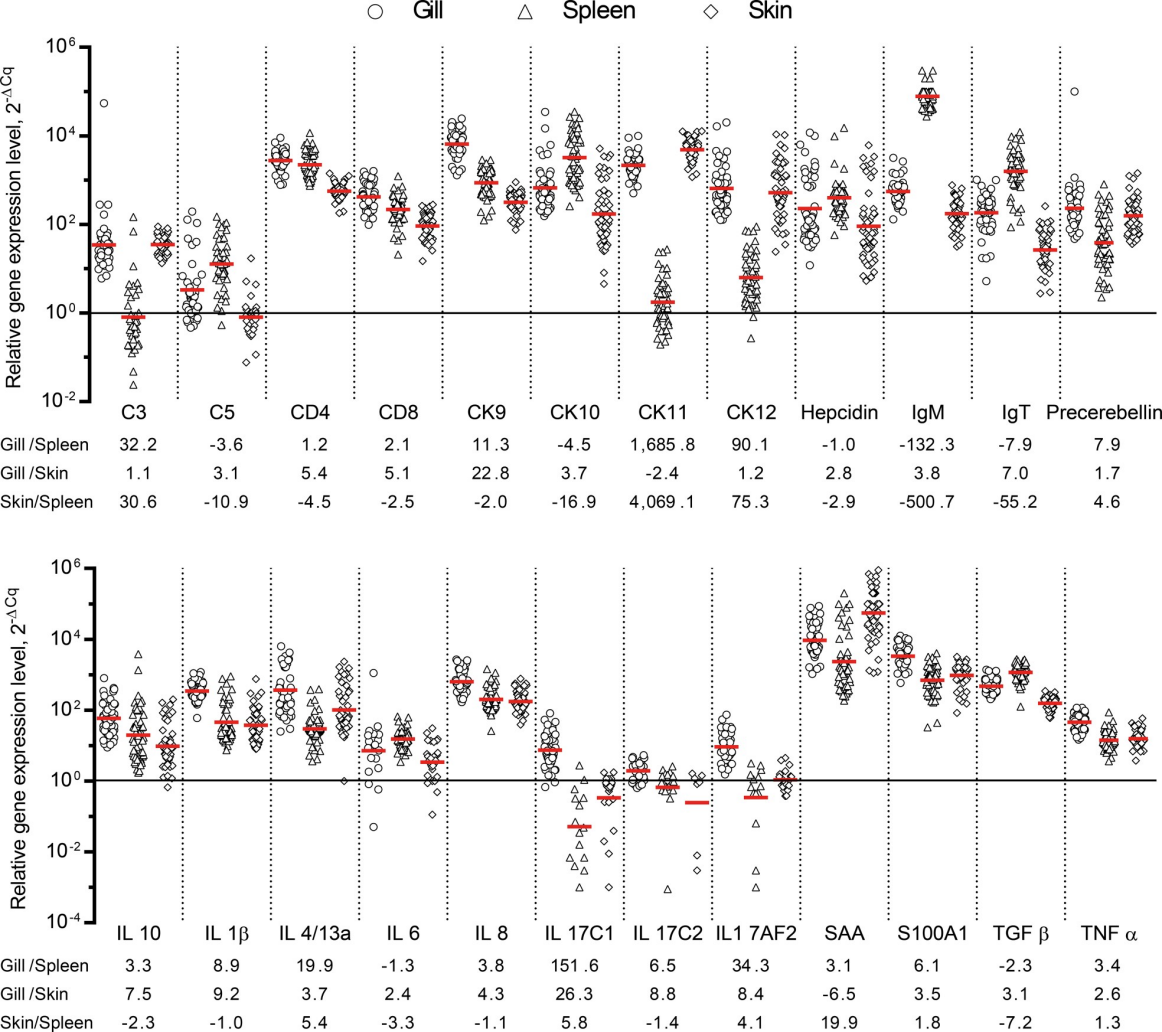
Overall expression levels of genes. The relative expression levels (both time points) were calculated as 2^-ΔCq^. The levels were normalized to the geometric mean of the least expressed gene (IL-17C2) (all tissues combined). All samples having valid Cq values are represented. Relative overall gene expression level (for each gene) between tissues is shown as the ratio (Gill/Spleen, Gill/Skin, Skin/Spleen).

We measured the expression of a total of 24 immune-related genes encoding cytokines (IL-1β, IL-4/13A, IL-6, IL-8, IL-10, IL-17/C1, IL-17/C2, IL-17A/F2, TGFβ, and TNFα), chemokines (CK9, CK10, CK11, and CK12), complement factors (C3 and C5), acute phase proteins (hepcidin, precerebellin, SAA, and S100A1), immunoglobulins (IgM and IgT), and cellular receptors (CD4 and CD8). The overall percentage of samples having a Cq value was 94% in gills compared to 89% in both skin and spleen. The expression profiles of the genes encoding CD4, IL-1β, IL-8, IL-17C1, SAA, S100A1, TGFβ, and TNFα from gill tissue at 8 dpi were presented in a previous work [12]. The expression level differed from gene to gene in all tissues examined (Fig 2) and the majority of genes showed altered expression after infection. Genes encoding complement (C3 and C5) and cytokines IL-17 showed low expression particularly in skin and spleen. A total of 22 genes had higher expression in gills compared to skin (the genes encoding CK11 and SAA had a higher transcript level in skin). Seventeen genes in gills showed higher expression compared to the corresponding genes in spleen, but genes encoding C5, CK10, hepcidin, IL-6, TGFβ, IgM and IgT were expressed at higher level in spleen. A total of 10 genes encoding C3, CK11, CK12, precerebellin, IL-4/13A, IL-17/C1, IL-17A/F2, SAA, S100A1, and TNFα were expressed at a higher level in skin compared to spleen, whereas 14 genes showed higher expression in spleen.

**Fig 3:**
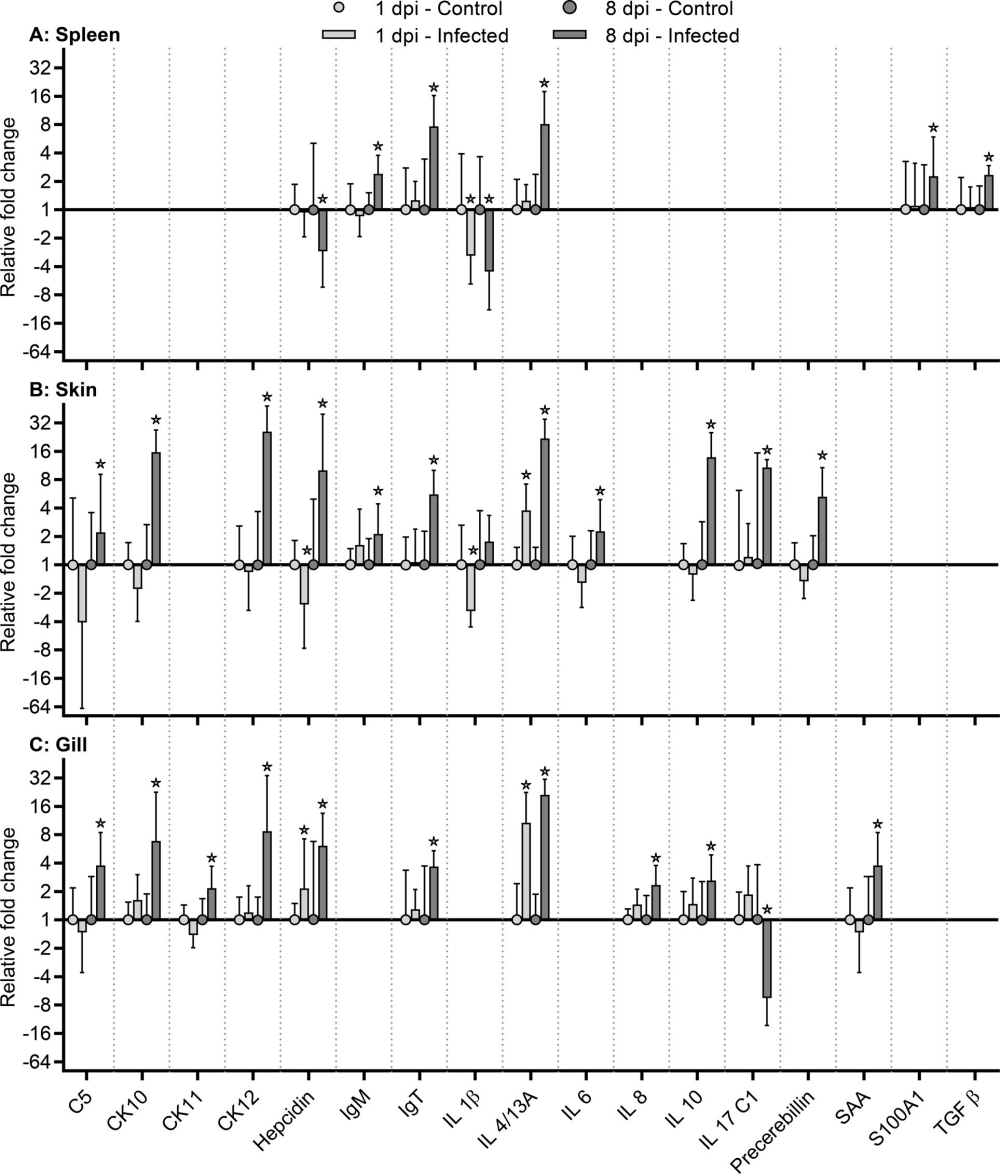
Gene expression analysis. Relative fold change was calculated as 2^-ΔΔCq^. Due to their exponential nature, the geometric mean and geometric standard deviation were used. The EF1α was used as the reference gene (housekeeper). Only significant results are shown. A comprehensive summary of the gene expression study, including all the genes investigated, is present as supplementary material S3 Table. *: p<0.05 (Student’s t-test), fold change >2.

**Fig 4:**
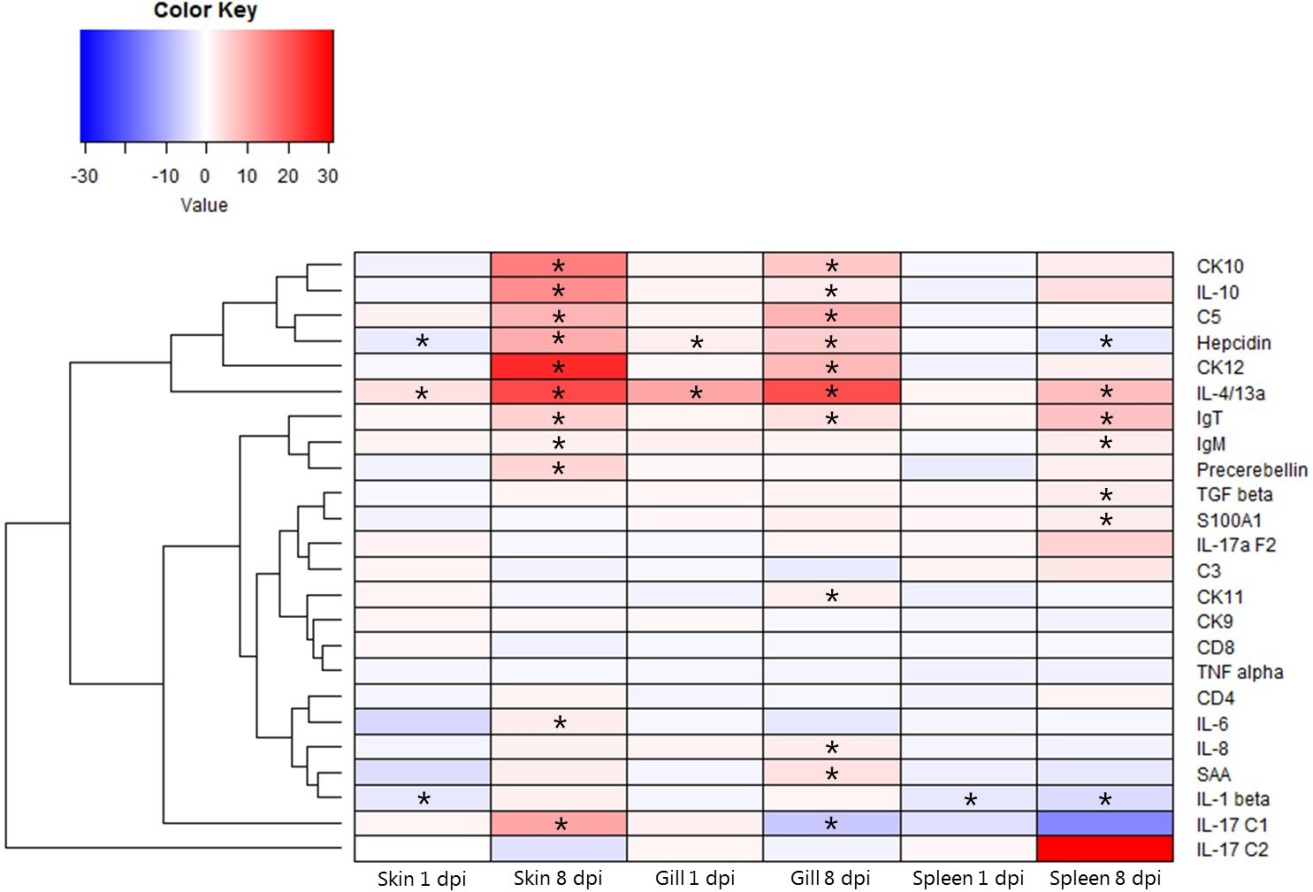
Heatmap showing the relative gene expression of immune associated genes in skin, gills, and spleen of rainbow trout, at different time points. Infected fish compared to time point control fish. Red and blue indicate up-regulation and down-regulation, respectively. *: significant different from control group, p<0.05. The hierarchical clustering orders the rows based on similarity of expression levels (log2 fold change). The dendrogram indicates both similarity and the order that cluster are formed.

Expression levels in skin, gills and spleen at different time-points were correlated as shown by significant positive correlations of 8 genes (encoding CK10, CK12, hepcidin, SAA, IL-4/13A, IL-17A/F2, IgM, IgT) in gills and skin at 1 dpi. Genes encoding CK12, SAA, and IgM also showed significant positive correlation between the two mucosal surfaces at 8 dpi. The expression of genes encoding C3, C5, hepcidin, precerebellin, SAA, S100A1, IL-10, CD4, CD8, IgT in gills was positively correlated with expressions in spleen at 1 dpi, while only CD4 was positively correlated at 8 dpi in between these tissues. A significant positive correlation of the IgM gene expression in skin and spleen was found at 1 dpi, while the IL-10 gene expression was negatively correlated at day 8 after infection (S2 Table). Merely a small number of genes showed a significant regulation at day 1 after infection, some of which were up-regulated and others were down-regulated. In contrast, the majority of investigated genes at day 8 post-exposure showed a significant up-regulation associated with infection (Figs 3 and 4).

#### Cytokines

The expression of the gene encoding IL-1β was significantly decreased in the spleen at both time points. Its expression was also significantly decreased in the skin at day 1 pi (but not regulated at day 8 pi) whereas no significant regulation was observed in gills at any time point. The gene encoding IL-6 was significantly up-regulated in the skin at day 8 pi, but not at day 1 pi. Expression of the IL-6 gene did not differ between gills and spleen. The gene encoding IL-17/C1 showed significant up-regulation in the skin of infected fish at day 8 pi contrasting the down-regulation found in gills of infected fish. No significant regulation of the IL-17/C1 gene was seen in any of the tissues at day 1 after infection and in spleen during the experiment. A significant regulation of the gene encoding IL-4/13A was seen both in gills and skin, both at day 1 and 8 pi, but it was only significantly increased at day 8 pi in spleen. The gene encoding IL-10 was also significantly up-regulated in skin and gills at 8 dpi but not at 1 dpi and in spleen (both time points). Expression of the TGFβ gene was only significantly increased in the spleen at day 8 post-infection and not regulated in skin and gills at any time point. No significant regulation of genes encoding IL-17/C2, IL-17A/F2, and TNFα was observed for any of the tissues during the infection.

#### Complement factors

Two genes encoding complement factors (C3 and C5) were analyzed in this study, but only the C5 gene showed a significant regulation after infection as it was significantly up-regulated in skin and gills at day 8 pi but not at day 1. No significant changes of the C3 and C5 gene expression were observed in the spleen.

#### Chemokines

The genes encoding chemokines CK10 and CK12 and IL-8 were significantly expressed in the gill and skin of infected fish at day 8 pi but no significant regulation was seen in the spleen during infection. Expression of the CK11 gene was significantly up-regulated in gills of infected fish at day 8 pi, whereas no regulation of CK9 and CK11 genes in examined skin and spleen tissues was recorded.

#### Acute phase proteins

Regulation of the gene encoding hepcidin exhibited higher variability than the other acute phase proteins (precerebellin and serum amyloid A SAA). A significant increase in expression of the hepcidin gene was observed in the gills of infected fish at all sampling points and in skin at day 8. However, it was down-regulated in skin at day 1 pi and in spleen day 8 pi. A significant increase in expression of the precerebellin gene was only found in the skin of infected fish at day 8 pi whereas this gene showed a stable expression in gills and spleen during infection. A significant upregulation of the gene encoding SAA was found in gills of infected fish at day 8 pi, but not activated in skin and spleen at all. Similar to the cytokine TGFβ gene, the expression of the gene encoding S100A1 (involved in neurotransmitter signaling) was merely significantly elevated in the spleen at day 8 after infection, and not regulated in skin and gills at any sampling point.

#### Immunoglobulins and cellular receptors

The expression of immunoglobulins was clearly increased in the central immune organ compared to both skin and gills. A significant increase of the immunoglobulin T (IgT) gene transcription in examined tissues was observed especially at day 8 pi. The gene encoding IgM was up-regulated at day 8 pi in spleen and skin but in gills merely a slight IgM gene regulation was indicated. Significant regulations (more than 2-fold) for the genes encoding T cell receptors CD4 and CD8 were not observed.

## Discussion

Rainbow trout responded, both at external surfaces and in the central immune organ spleen, to infection with the ciliated protozoan *I*. *multifiliis* by regulating both innate and adaptive immune genes. The reaction was correlated to the increasing severity of infection as the parasites increased their volume from theront size (30–40 μm) to maximum trophont size (> 500 μm) during the course of infection. In addition to the increased size of the parasite it is suggested that the elevated feeding and presentation of antigens from the growing trophont stimulate the infected tissue. Thus, *I*. *multifiliis* ciliates possess protein structures termed immobilization antigens (I-antigens) covering up to 60% of their surface [14]. Merely a few immune-related genes were regulated at day 1 pi compared to day 8 pi, at which time point these immune parameters dramatically changed. Regulation of immune genes in affected host organs have previously been reported [7, 15] but the present study demonstrated that gills (harbouring a higher infection) responded significantly stronger compared to skin having a lower parasite burden (as judged from the I-antigen expression). Antigen presenting cells in rainbow trout gills are sentinels for external stimuli [16] and gills may in general respond stronger to stimulation compared to skin. Exposure of trout to the bacterial pathogen *Yersinia ruckeri* elicited higher immune gene expression in gills [17] corresponding to findings in other fish models (non-infected) including miiuy croaker [18] and Japanese pufferfish [19].

In addition, the response in the spleen, which did not harbor any infection at all, was primarily concentrated about genes encoding immunoglobulins (IgM and IgT) and cytokines associated with humoral immunity (IL-4/13). This indicates that local protective responses raised in the mucosal surfaces (gills, skin) can transmit signals to central immune organs establishing a systemic response few days after exposure. Candidates involved in signaling between gills/skin and the spleen may be genes encoding chemokines CK10 and CK12 and the cytokine IL-4/13A. These genes were highly upregulated in gills and skin at day 8 concomitant with immunoglobulin gene upregulation in spleen. Despite the importance of a systemic protection based on mainly immunoglobulin IgM and IgT production [8, 20] a local reaction involving a series of innate factors is associated with both the primary and secondary response to the infection [4].

In this context it is noteworthy that genes expressed both in gills and skin encoded chemokines (CK10, CK12), APPs (hepcidin, SAA), cytokines (IL-4/13A, IL-17A/F2), and immunoglobulins (IgM, IgT) which indicate their role in the barrier function (early and late) against penetrating pathogens [21]. Also at the systemic level immune genes encoding complements (C3, C5), APPs (hepcidin, precerebellin, SAA, S100A1), cytokines IL-10, cell receptors (CD4, CD8), were expressed although at a lower level compared immunoglobulin genes. However, again the extrahepatic expression of complement varied considerably between tissues, showing that complement factor genes were mainly expressed in gills.

Some cytokine genes were mainly down-regulated including IL-1β, an important pro-inflammatory cytokine in fish [13]. Although this cytokine may play a role in the initiation of the early immune response and has multiple effects on gene expression during inflammation [22], IL-1β expression was likely suppressed in rainbow trout at later stages of the infection. Depression of pro-inflammatory cytokine production in rainbow trout macrophages infected with *Renibacterium salmoninarum* was previously demonstrated [23] and it cannot be excluded that a corresponding regulations is associated with *I*. *multifiliis* infection and at least partly connected to cortisol elevation [24–26]. Also TNFα, considered as an important component in the inflammatory response in fish [27] and activated in rainbow trout after i.p. injection of live theronts of *I*. *multifiliis* [15], was not induced by infection. Genes encoding cytokines IL-6 and IL-17 C1 were the only of pro-inflammatory cytokine genes slightly up-regulated in the skin at day 8 pi corresponding to the *Ichthyobodo* induced skin response after severe epidermal emaciation [28]. At the same time suppression of IL-17 C1 was found in gills at day 8 even though it showed a weak up-regulation at early of infection, which will frame the different responsiveness of gills and skin.

The complement system [29] is an essential part of the innate immune system [30] in alerting the host of the presence of potential pathogens [31] and playing a crucial role in the response or resistance against Ich [5, 32, 33]. In the present study mainly complement factor C5 played a role in the physical barriers skin and gills corresponding to previous studies of this parasite-host model [6, 7, 15] and against the bacterial pathogen *Yersinia ruckeri* [34]. C3 expression was less prominent in this work contrasting previous work showing high expression in liver, head kidney, skin, gill, and spleen [5–7, 15].

Chemokines are secreted immune factors attracting a diverse set of effector leukocytes to inflammatory sites [35]. The chemokines IL-8, CK10, CK11, and CK12 were significantly regulated in skin and gills at day 8 after infection and as previously shown the expression of chemokine genes in response to infection may differ between tissues [36–38]. In general regulation of chemokine genes CK10, CK11, and CK12 occurs during viral infection of rainbow trout [38, 39] and as shown by Munoz-Atienza et al. [40] CK11 may have direct antiparasitic effects and act as the first line of defense against infection.

The present study showed that some of the regulating or immune-modulatory cytokines such as IL-10 and TGFβ were activated. This would provide a control of pro-inflammatory actions during infection [41] limiting the potentially injurious effects elicited by excess inflammatory reactions [42]. Regulating cytokines such as IL-4/13A can suppress pro-inflammatory cytokine genes (such as IL-1 and TNF), and at the same time direct adaptive immune pathways involving B lymphocytes [43–46]. Interleukin IL-4 and IL-13 are closely related cytokines important for Th2 responses especially for defense against parasites [47].

The expression of acute phase response genes may have been induced by plasma-borne signals [48] some of which may stimulate leukocytes and hepatocytes to release acute phase proteins (APPs) into the bloodstream [29, 49]. Genes encoding serum Amyloid A (SAA), precerebellin, and Hepcidin were slightly elevated after infection in the present study. SAA has been suggested to directly influence the parasitic ciliate *Ichthyophthirius multifiliis* [6, 50] and the flagellate *Ichthyobodo necator* [28]. Expression of precerebellin which is part of the acute phase response in rainbow trout [51, 52] was elevated significantly in the skin, but involved to a lower extent in the gills and spleen. The link between inflammation and production of hepcidin has been demonstrated by previous studies [53–56]. During inflammation, the cytokine IL-6, a mediator of acute phase reaction [57], induces production of hepcidin [29] also in extrahepatic organs such as gills and skin as judged from the present work.

Activation of the gene encoding S100A1 in the spleen is less clear but it is indicated that it is part of the inflammatory response as suggested by zebrafish studies [58]. However, other functions may be exerted by the molecule S100A1 as it is a member of the S100 protein family expressed only in vertebrates [59], is calcium-binding, involved in plasma membrane transport [60] and essential in the acute response to hemodynamic stress [61]. In addition, in mammals the extracellular S100 proteins exert regulatory activities on immune cells, thereby participating in innate and adaptive immune responses, cell migration and chemotaxis, tissue development and repair, and leukocyte and tumor cell invasion [59].

## Conclusion

The establishment of an immune response towards I. *multifiliis* occurs both at a local and a systemic level as seen by expression of immune genes in all examined tissue. Expression of immune genes in gills and skin followed the same pattern although gills showed a higher expression level suggesting that gills are more exposed or play a role as sentinels for external stimulation including exposure to pathogens. Despite absence of pathogens in the spleen at any time point the spleen showed elevated immune gene expression especially with regard to immunoglobulin production which suggests that signaling, associated with chemokines and cytokines, from infected mucosal surfaces may reach the spleen and establish a systemic protective response.

## Supporting information

S1 Table Primers and probes used for qPCR assays. (PDF)

S2 Table Correlation among the gene expression levels in gills, skin, and spleen at different time-points. Correlation value and p-value are provided.
(XLSX)

S3 Table A comprehensive summary of the gene expression study including all investigated genes. (XLSX)
