# Supplementary material for: Differential immune gene response in gills, skin, and spleen of rainbow trout Oncorhynchus mykiss infected by Ichthyophthirius multifiliis
Source: PLoS One. 2019 Jun 20;14(6):e0218630. doi: 10.1371/journal.pone.0218630 (PMC6586319; doi:10.1371/journal.pone.0218630)
Supplement: S1 Table — (PDF) [file pone.0218630.s001.pdf]

S1 Table. *Primers and probes used for qPCR assays.*

Gene	Gene ID	Accession No.	Forward primer	Reverse primer	Probe
Cytokines					
IL-1 beta	100136024	AJ223954	ACATTGCCAACCTCATCATCG	TTGAGCAGGTCCTTGTCCTTG	CATGGAGAGGTTAAAGGGTGGC
IL-4/13a	100653462	AB574337	ATCCTTCTCCTCTCTGTTGC	GAGTGTGTGTGTATTGTCCTG	CGCACCGGCAGCATAGAAGT
IL-6	100136689	DQ866150	ACTCCCCTCTGTCACACACC	GGCAGACAGGTCCTCCACTA	CCACTGTGCTGATAGGGCTGG
IL-10	100136835	AB118099	CGACTTTAAATCTCCCATCGAC	GCATTGGACGATCTCTTTCTTC	CATCGGAAACATCTTCCACGAGCT
IL-17 C1	100462681	CAW30792	CTGGCGGTACAGCATCGATA	GAGTTATATCCATAATCTTCGTATTCGGC	CGTGATGTCCGTGCCCTTTGACGATG
IL-17 C2	100462682	CAW30793	CTGGCGGTACAGCATCGATA	CAGAGTTATATGCATGATGTTGGGC	CGTGGTGTCCAGGCCCTTTAATGATG
IL17 A/F2	100136642	AJ580842	TCAAAAGCAACGTGTCGAAG	TCCCTCTGATTCCTCTGTGG	TATGCTGCTGGGCCTGACCA
TGF beta	100136774	X99303	TCTGAATGAGTGGCTGCAAG	GGTTTCCCACAATCACAAGG	CTGGAGAGGAGCAGGGATTCCAAT
*TNF alpha**	100136034<br>100136064	AJ277604<br>AJ401377	GGGGACAAACTGTGGACTGA	GAAGTTCTTGCCCTGCTCTG	GACCAATCGACTGACCGACGTGGA
Complement factors					
C3	100135918	AF271080	ATTGGCCTGTCCAAAACACA	AGCTTCAGATCAAGGAAGAAGTTC	TGGAATCTGTGTGTCTGAACCCC
C5	100135962	AF349001	TGGCAAGGACTTTTTCTGCT	AGCACAGGTATCCAGGGTTG	CTGGCAGGGATTGCATCAAATC
Chemokines					
IL-8	100136039	AJ279069	AGAATGTCAGCCAGCCTTGT	TCTCAGACTCATCCCCTCAGT	TTGTGCTCCTGGCCCTCCTGA
CK9	NI	CA378686	TGTACCTCACCATGGCTCAA	GCATGATCACAACAGCCTTG	GAAGCACGTTGCTGGCCTGA
CK10	NI	CA361535	ATTGCCAAGATCCTCTTCTGTGTTC	CCTGAGGCTGGTAACCTATGACAAC	CAGGGCCAGATGGTGATGGA
CK11	NI	BX072681	TTGGGACATCCAGGAACATT	ACAACAGCGTCGATCTCACA	GATGTCCAACACAACCATGGT
CK12	NI	CA346383	AACACTCTGCTTGGGCTACG	GAGGGAAACGGATGTCAGTG	CGCAGCAGCATTCTCAGAGG
Acute phase protein					
SAA	100136775	X99385	GGGAGATGATTCAGGGTTCCA	TTACGTCCCCAGTGGTTAGC	TCGAGGACACGAGGACTCAGCA
Hepcidin	100135935	AF281354	GAGGAGGTTGGAAGCATTGA	TGACGCTTGAACCTGAAATG	AGTCCAGTTGGGGAACATCAACAG
Precerebellin	100135893	AF192969	TGGTGTTGCTTTGCTGTTGT	GCCACTTTTGGTTTGCTCTC	ATGGTTGAGACTCAGACGGAGAGTG
S100A1	110510474	BT073924	GAGCTCAAGGACCTGATGGA	TCCCCATCTTCTTCTTGTGC	GCCTGTCCATCGCCTGTGAA
Cell receptors					
CD4	100136285	AY973028	CATTAGCCTGGGTGGTCAAT	CCCTTTCTTTGACAGGGAGA	CAGAAGAGAGAGCTGGATGTCTCCG
CD8 alpha	100135889	AF178054	ACACCAATGACCACAACCATAGAG	GGGTCCACCTTTCCCACTTT	ACCAGCTCTACAACTGCCAAGTCGTGC
Immunoglobulins					
IgM	NI	S63348	CTTGGCTTGTTGACGATGAG	GGCTAGTGGTGTTGAATTGG	TGGAGAGAACGAGCAGTTCAGCA
IgT	NI	AY870265	AGCACCAGGGTGAAACCA	GCGGTGGGTTCAGAGTCA	AGCAAGACGACCTCCAAAACAGAAC
Parasite gene					
I-antigen (Ich)	NI	AF324424	CTGCTGGCACTCTTGTTAC	CATTCAGTACATGTATCAGTACC	ACGATTGCCCTGCTGGTACA
Reference gene					
EF1-alpha	100136004	AF498320	ACCCTCCTCTTGGTCGTTTC	TGATGACACCAACAGCAACA	GCTGTGCGTGACATGAGGCA

**primer recognizes two isoforms of TNF alpha; NI: not identified.*
